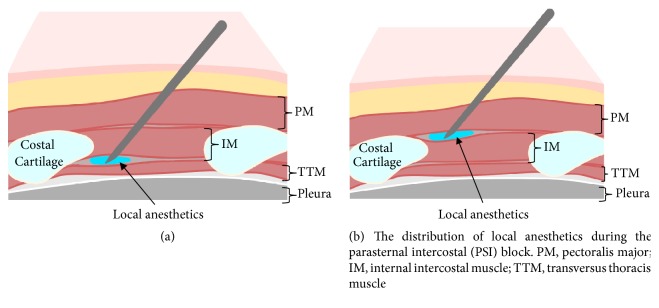# Transversus Thoracis Muscle Plane Block

**DOI:** 10.1155/2019/1716365

**Published:** 2019-07-07

**Authors:** Satoru Fujii, Ranjana Bairagi, Matthew Roche, Jian Ray Zhou

**Affiliations:** ^1^Department of Anesthesia & Perioperative Medicine, London Health Sciences Centre, Western University, 339 Windermere Rd, London, ON, Canada N6A 5A5; ^2^Department of Anesthesia & Perioperative Medicine, London Health Sciences Centre, Schulich School of Medicine and Dentistry, Western University, 339 Windermere Rd, London, ON, Canada N6A 5A5; ^3^Schulich School of Medicine and Dentistry, Western University, 339 Windermere Rd, London, ON, Canada N6A 5A5; ^4^Anesthesiology, Perioperative and Pain Medicine, Rockyview General Hospital, Cummings School of Medicine, University of Calgary, 7007 14 St SW, Calgary, AB, Canada T2V 1P9

## Abstract

The transversus thoracis muscle plane block (TTP) block is a newly developed regional anesthesia technique which provides analgesia to the anterior chest wall. Since its introduction, this technique has been utilized for a wide range of surgical procedures as well as nonsurgical indications. Current evidence suggests that the TTP block provides effective analgesia for breast and cardiac surgeries, cardiac device implantation, pericardiocentesis, and acute and chronic pain management. To date, no major complications have been reported. Currently there is an urgent need to standardize the nomenclature of this technique to facilitate accurate communication amongst care providers, researchers, and authors. In this review, we describe the TTP block technique, review the indications and available evidence in clinical practice, and discuss alternative blocks and future prospects.

## 1. Introduction

The transversus thoracis muscle plane block (TTP) block is a newly developed regional anesthesia technique which provides analgesia to the anterior chest wall. First described by Ueshima et al. in 2015 [1], the TTP block is a single-shot nerve block that deposits local anesthetic in the transversus thoracis muscle plane between the internal intercostal and transversus thoracis muscles. In the original ultrasound-guided cadaveric study, the TTP block was found to cover the T2-T6 intercostal nerves [2]. The anterior branches of these intercostal nerves dominate the sensory innervation of the internal mammary region, suggesting this new technique had potential to provide analgesia for surgery of the anterior chest wall. In a subsequent study, Ueshima et al. showed that the TTP block in combination with bilateral pectoral nerve blocks (Pecs II) enabled successful bilateral breast cancer resections without the aid of general anesthesia [1]. The success of this technique for breast resections spurred investigation for applications in other surgeries, such as in cardiovascular procedures.

Cardiac surgical patients often experience significant postoperative pain at the median sternotomy site. Mueller et al. [3] showed the location of maximal postoperative pain from postoperative day (POD) 1 to 7 is the sternal region, and Lahtinen et al. [4] found 75% of patients still complained of chest pain on POD 4. Acute postoperative pain is associated with sympathetic activation, hemodynamic instability, pulmonary complications, and delirium [5–7]. Furthermore, ineffective treatment of acute postoperative chest pain can precipitate persistent postopertaive pain. In a systematic review with meta-analysis, Guimarães-Pereira et al. found that persistent postoperative pain affected 37% of cardiac surgical patients in the first 6 months and continued to affect 17% for more than 2 years after surgery [8]. Contemporary studies included in the meta-analysis showed a higher incidence of persistent postoperative pain than previously noted [8, 9]. A systematic review by Bignami et al. [9] found that opioids are the primary analgesic modality for treating postoperative pain after cardiac surgery; however, opioids are not always adequate for analgesia and cause a myriad of adverse effects. Regional anesthesia techniques such as neuraxial and paravertebral techniques are not routinely utilized in this patient population due to concerns of complications after heparinization and coagulopathy [9]. Due to the more superficial anatomy of the TTP block, the risk of bleeding complications is reduced. In fact, Ueshima et al. successfully administered continuous TTP blocks in a patient undergoing cardiac surgery [6]. The TTP block has also been successfully used in a multitude of surgical and nonsurgical applications recently [2, 6, 8–10].

This review will describe the indications, techniques, and applications for the TTP block along with alternative regional modalities.

## 2. Technique

**Figure 1:**
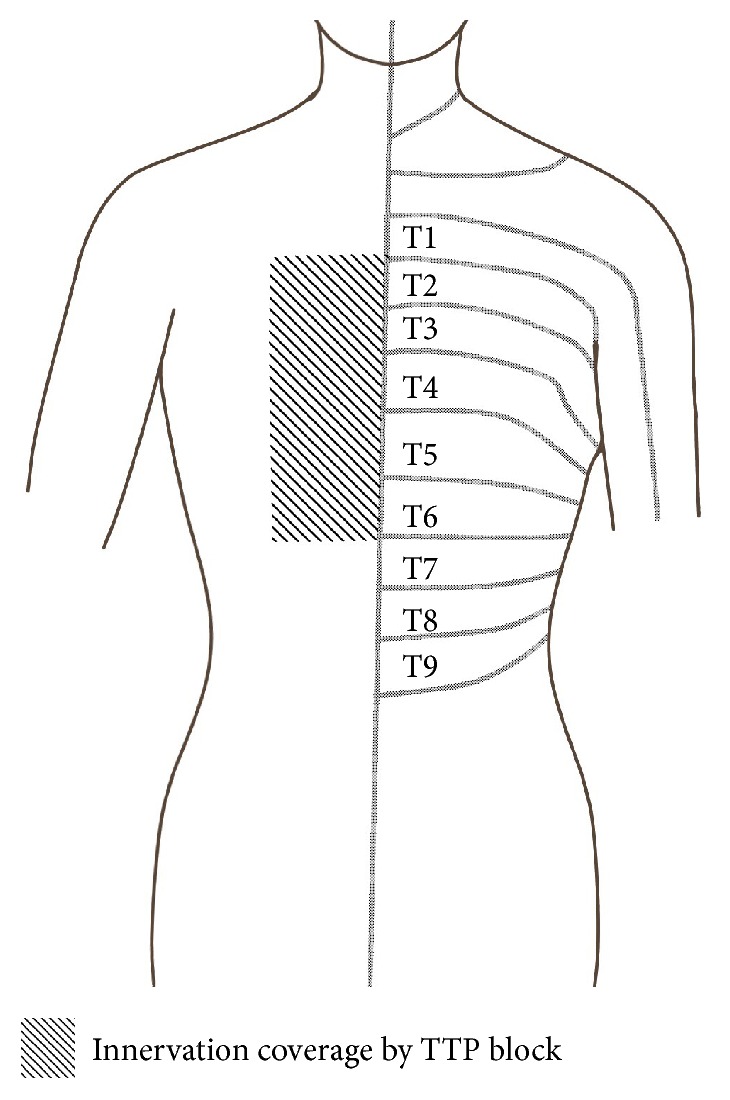
The innervation coverage of the TTP block [11, 20]. The coverage would vary depending on the volume of injectate and the injection site. TTP, transversus thoracis muscle plane.

**Figure 2:**
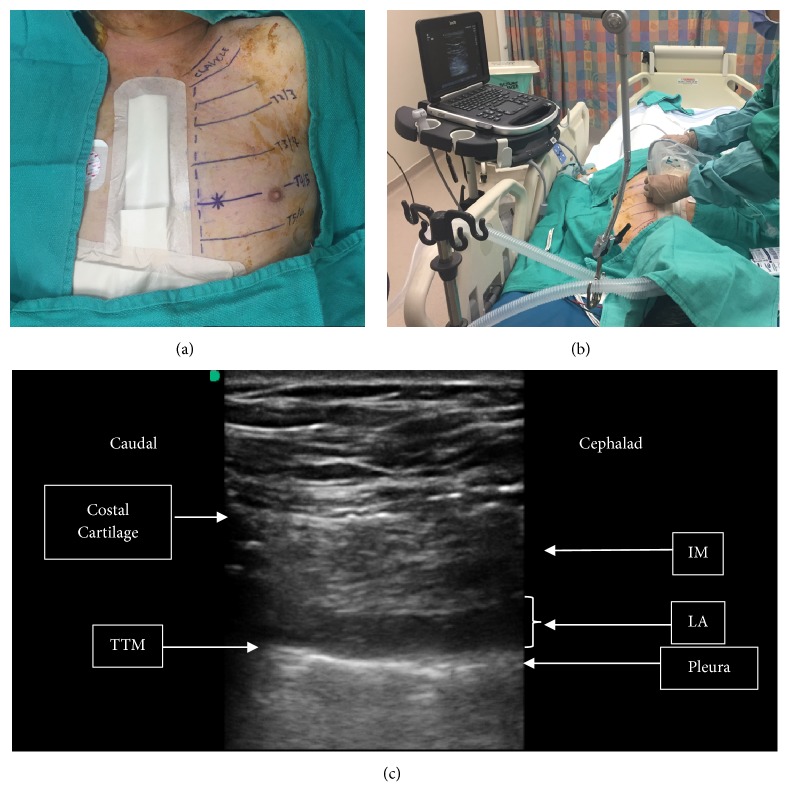
Patient positioning and ultrasound images in the TTP block. (a) Anterior chest wall of patient after cardiac surgery. The thoracic intercostal spaces are marked. The injection site is indicated by an asterisk. (b) The operator stands beside the patient with ultrasound machine on opposite side. The ultrasound probe is placed longitudinally to the sternum. (c) Ultrasound image after TTP block. The local anesthetic spreads uniformly between the IM and TTM. The pleura is displaced downwards. IM, internal intercostal muscle; TTM, transversus thoracis muscle; LA, local anesthetics.

The TTP block technique has been described in the literature with some variation depending on the center and the operator [1, 11–18]. The area of analgesic coverage by the TTP block is depicted in Figure 1. At our center, the TTP block is performed under ultrasound (US) guidance using a high-frequency linear transducer. First, the ultrasound probe is placed in the longitudinal plane 1 cm lateral to the sternal border. Next, the T4-T5 intercostal space is identified under US (Figures 2(a) and 2(b)). A parasternal sagittal view of the internal intercostal muscle and the transversus thoracis muscle between the 4th and 5th rib is visualized above the pleura. A 22-gauge, 80-mm needle is inserted in-plane to the transducer with bevel up until the tip of the needle is located in the transversus thoracis muscle plane between the internal intercostal and transversus thoracis muscles (Figure 2(c)). After excluding intravascular and intrapleural placement, local anesthetic is administered in 5 mL aliquots with intermittent aspiration. Twenty milliliters of 0.3% (if patients weight<75kg) or 0.5% ropivacaine (if patients weight≥75kg) is administered bilaterally. The type, concentration, and dosage of local anesthetic have not been standardized, and further studies are needed to provide more robust data regarding the optimal injectate.

Murata et al. reported [19] that the downward movement of the pleura is a good indication of successful injection into the TTP, whereas the spread of injectate above the costal cartilage indicates the injection is above the internal intercostal muscle. After block completion, the patient is closely monitored for 20 minutes for local anesthetics toxicity, hemodynamic instability, and allergic reaction. The time required for bilateral block performance ranges from 10 to 15 minutes.

Another group has published a letter [20] reporting better spread of local anesthetic when the ultrasound is positioned in the sagittal plane. At our center, we are performing this block for patients undergoing median sternotomy for cardiac surgery. With the ultrasound probe in the longitudinal position (Figure 2(c)), we can site the injection closer to the sternum, our target of interest.

Regarding the best intercostal space for this block, a study involving 10 healthy volunteers demonstrated that injection at the T4-T5 intercostal space improved the spread of local anesthetic compared with injection at T3-T4 [15]. In our cadaver study [11] injection of local anesthetic at T4-T5 resulted in spread from the manubrium to the T5-T6 intercostal space.

As of April 2019, no randomized controlled trials have been conducted investigating the use of the TTP block in the pediatric population or the use of a continuous regional infusion. One letter [17] reported a TTP catheter strategy employing intermittent boluses of 10 mL levobupivacaine 0.1% on each side every hour in addition to demand doses of 3 mL levobupivacaine 0.1% every 30 min for two postoperative days. Further clinical trials are warranted to study these administration approaches.

## 3. Current Indications and Available Evidence

No clear indication exists for the TTP block; however, a number of case reports have been published describing novel applications of this block. The available literature demonstrates the use of TTP blocks in breast resection surgery, subcutaneous internal cardiac defibrillator placement [10], and pericardial effusion drainage [21]. Other applications include acute pain management associated with sternal fractures [22] and chronic pain treatment for postthoracotomy internal mammary pain syndrome [12].

### 3.1. Median Sternotomy

Ueshima et al. [17] described the successful use of bilateral continuous TTP blocks for perioperative pain management in two surgical patients requiring median sternotomy. The first patient underwent aortic valve replacement, and the second patient received a thymectomy. In both cases, the TTP blocks were performed while the patient was under general anesthesia. With ultrasound guidance, 40 mL of 0.375% levobupivacaine was injected bilaterally into the fascial plane between the transversus thoracis muscle and the intercostal muscle at the fourth and fifth intercostal space. Subsequently, a continuous infusion of 0.1% levobupivacaine at 10 mL per hour per side was administered through catheters inserted at each injection site. Additional demand doses of 3 mL of 0.1% levobupivacaine were available every 30 minutes. Postoperatively, both patients required no additional analgesia. This report showed bilateral continuous TTP blocks alone provided sufficient perioperative analgesia for median sternotomy.

### 3.2. Breast Surgery

The TTP block has also been found to provide adequate surgical analgesia for patients requiring breast resection when combined with the Pecs II block in 3 cases [1]. The TTP blocks were performed under US guidance using 15 mL of 0.15% levobupivacaine injected between the third and fourth ribs. The Pecs II block was performed by administering 10 mL of 0.15% levobupivacaine between the pectoralis major and minor muscle at the third rib. A further 20 mL was injected between the pectoralis minor and serratus muscles at the fourth rib. All 3 cases of breast cancer resection were then successfully completed under propofol or dexmedetomidine sedation. These patients had uneventful postoperative courses and were discharged home without any analgesic drugs. For breast surgery, the TTP block is insufficient as a sole technique, and the addition of the Pecs II block is necessary.

### 3.3. Surgery with Thoracotomy

A number of analgesic approaches have been used in robotic-assisted mitral valve surgery, including intrathecal opioids, local anesthetic infiltration, thoracic epidural catheter placement, intercostal nerve block, and paravertebral nerve block [23]. Other regional techniques include US-guided pectoralis blocks (Pecs I and Pecs II) and the serratus plane (SP) block. The Pecs II block covers dermatomes T2-T4, with variable spread to T6. The SP block has been used to treat thoracotomy pain and pain associated with multiple rib fractures. There are currently no studies published on the use of pectoralis, serratus plane, or TTP blocks in robotic-assisted or minimally invasive cardiac surgery. Theoretically, when the incision extends beyond the coverage of the Pecs I, Pecs II, and SP blocks, supplementing with the TTP block may be beneficial.

### 3.4. Implantable Cardioverter-Defibrillator (ICD) Implantation

Two case reports have described the use of the TTP block for subcutaneous implantation of ICD. Bhatt et al. used a combination of serratus plane and TTP block [10], while Ueshima et al. employed the TTP block with the thoracic paravertebral block [24]. Both cases did not require any additional intra- or postoperative opioids. Bhatt et al. performed the SP block using 20 mL of 0.25% bupivacaine injected between the latissimus dorsi and serratus anterior muscle. Similarly, the TTP block was performed using 20 mL of 0.25% bupivacaine deposited between the internal intercostal and transversus thoracis muscle. The TTP block covers the anterior cutaneous branches of the intercostal nerves which are spared by the SP block. A combination of TTP and SP blocks is required for ICD implantation.

### 3.5. Miscellaneous Procedures

The TTP block has also been exclusively used for pericardiocentesis without general anesthesia [21]. One case involved an 81-year-old patient who developed cardiac tamponade five days after undergoing emergency Percutaneous Coronary Intervention (PCI) for acute myocardial infarction. A single left-sided TTP block was performed with 20 mL of 0.375% levobupivacaine at the level of the fourth and fifth rib. Pericardial drainage was performed via subxiphoid approach.

A second case involved a 60-year-old patient who required pericardiocentesis after type A aortic dissection. The patient received bilateral TTP blocks, each with 20 mL of 0.375% levobupivacaine. Pericardial drainage was successfully performed 40 minutes later without general anesthesia. TTP blocks alone provide adequate anesthesia for pericardial effusion drainage via subxiphoid approach.

### 3.6. Acute and Chronic Pain

The TTP block has played a role in both acute and chronic pain management. Thomas et al. described an US-guided parasternal block for pain relief after sternal fracture sustained from motor vehicle collision [22]. In their report, the parasternal block technique and injection site are identical to the TTP block. Initially, the patient complained of 4 out of 10 chest pain despite around the clock intravenous diclofenac 75 mg, paracetamol, and morphine. Subsequently, the patient developed respiratory acidosis from shallow breathing and splinting. A parasternal (TTP) block was performed with 16 mL of 0.75% ropivacaine injected bilaterally at the lateral border of the sternum. Ten minutes after injection, the patient was pain free at rest and on sternal pressure.

Piraccini et al. described the use of a different parasternal block with the TTP block to treat a patient suffering from postthoracotomy internal mammary pain syndrome ten months after surgery [12]. Pecs blocks do not provide analgesia to the internal mammary region, whereas the parasternal and TTP blocks do. Three weekly US-guided injections of ropivacaine 40 mg and triamcinolone 20 mg (in normal saline 20 mL for hydrodissection of the involved fascial planes) were administered. The parasternal block was performed between the pectoralis major and internal intercostal muscles over the 2nd and 4th rib, 2 cm lateral to sternum. The TTP block was performed between the transversus thoracis muscle and the intercostal muscle at the 4th rib, 5 cm lateral to the sternum. The patient reported immediate and complete resolution of pain that was maintained for 3 months after the injections. Although parasternal intercostal blocks may reduce acute postoperative pain, there is currently no evidence for their efficacy in preventing chronic poststernotomy pain [25]. For acute sternal fracture analgesia, TTP blocks alone appear to be adequate. Treatment of postthoracotomy pain syndrome may require the combination of TTP and parasternal blocks.

## 4. Complications

TTP block related complications can include local anesthetic toxicity, vascular injury, pneumothorax, failed block, and anaphylactic shock. The vulnerable structures adjacent to the injection site include the internal thoracic artery and vein, pleura, pericardium, and intercostal artery and vein. Ueshima et al. [26] monitored 299 patients who received TTP blocks and found only 2 patients developed superficial infection around the injection site.

When we conducted our feasibility study for TTP block administration in the cardiac surgery intensive care unit [27], none of the 9 patients who underwent cardiac surgery and received the TTP block experienced a block related complication. Further studies with larger sample sizes are warranted to investigate the rate of these rare complications.

The efficacy of the TTP block can be compromised by previous surgery in the TTP plane. We performed a cadaver study [11] looking at local anesthetic spread after TTP block in a patient who had previously undergone internal thoracic artery harvest. Tissue disruption and scar remodeling from surgery led to nonuniform spread of local anesthetic in the transversus thoracis plane, which could potentially lead to a failed or inadequate block.

## 5. Alternative Techniques

Ohgoshi et al. [28] recently reported that the transversus thoracis muscle is difficult to visualize under US. Since the internal thoracic artery is in the vicinity of the TTP block target, their group suggested a meticulous needling technique is required to perform this block. Alternatively, they recommended the parasternal intercostal nerve block (PSI block). For the PSI block, local anesthetic is injected into the plane between the pectoral major and intercostal muscles (Figure 3(b)). Since the injection plane is more superficial, the risk of pleural and arterial puncture is lower compared to the TTP block. The local anesthetic exerts its effect on the anterior branches of the intercostal nerves as they penetrate through these two muscle layers.

De la Torre et al. [29] reported successful use of the pecto-intercostal fascial block (PIFB) for breast surgery, which technically is identical to the PSI block. Victor Liu et al. [30] also used the PIFB for cardiac surgery. In their case report, the PIFB provided immediate and sustained pain relief after cardiac surgery; however, 3 needle insertions on each side were required to cover 5 interspaces. These authors postulate the PIFB is a superficial block and associated with less risk of arterial and pleural puncture. Currently, no studies have compared the TTP block to the above-mentioned alternatives in a clinical trial. If the efficacy of these blocks prove to be equal, a more superficial block would likely be safer.

## 6. Future Directions

At present, nomenclature for field blocks of anterior intercostal nerve branches are not standardized. The TTP and parasternal blocks [22] are essentially the same technique (Figure 3(a)). The PSI block [28] and PIFB [29] also refer to identical techniques (Figure 3(b)). Furthermore, the TTP block itself has multiple names, such as the transversus thoracic plane block [2], transversus thoracic muscle plane block [19], and transversus thoracis muscle plane block [27]. Further clarification and standardization of the nomenclature are needed to facilitate accurate communication amongst care providers, researchers, and authors.

These novel regional analgesic techniques appear very effective, promising, and safe when performed with ultrasound guidance. Moreover, recently published feasibility study by Fujii et al. [27] shows the potential benefit and feasibility of this technique in the cardiac surgery setting. As previously stated by other authors, randomized controlled trials are necessary to explore the utility of this technique for different surgical applications [19, 27].

## Figures and Tables

**Figure 3:**